# Neuroplasticity in Cholinergic Projections from the Basal Forebrain to the Basolateral Nucleus of the Amygdala in the Kainic Acid Model of Temporal Lobe Epilepsy

**DOI:** 10.3390/ijms20225688

**Published:** 2019-11-13

**Authors:** Ítalo Rosal Lustosa, Joana I. Soares, Giuseppe Biagini, Nikolai V. Lukoyanov

**Affiliations:** 1 Clinical and Experimental Medicine PhD Program, University of Modena and Reggio Emilia, 41125 Modena, Italy; italo.rosal@gmail.com; 2 Instituto de Investigação e Inovação em Saúde, Universidade do Porto, 4200-135 Porto, Portugal; joana.isa.soares@gmail.com; 3 Instituto de Biologia Molecular e Celular da Universidade do Porto, 4200-135 Porto, Portugal; 4 Departamento de Biomedicina, Faculdade de Medicina da Universidade do Porto, 4200-319 Porto, Portugal; 5 Programa Doutoral em Neurociências, Universidade do Porto, 4200-319 Porto, Portugal; 6 Department of Biomedical, Metabolic and Neural Sciences, University of Modena and Reggio Emilia, 41125 Modena, Italy; 7 Center for Neuroscience and Neurotechnology, University of Modena and Reggio Emilia, 41125 Modena, Italy

**Keywords:** acetylcholine, basolateral nucleus of amygdala, basal forebrain, kainic acid, neuronal plasticity, temporal lobe epilepsy

## Abstract

The amygdala is a cerebral region whose function is compromised in temporal lobe epilepsy (TLE). Patients with TLE present cognitive and emotional dysfunctions, of which impairments in recognizing facial expressions have been clearly attributed to amygdala damage. However, damage to the amygdala has been scarcely addressed, with the majority of studies focusing on the hippocampus. The aim of this study was to evaluate epilepsy-related plasticity of cholinergic projections to the basolateral nucleus (BL) of the amygdala. Adult rats received kainic acid (KA) injections and developed status epilepticus. Weeks later, they showed spontaneous recurrent seizures documented by behavioral observations. Changes in cholinergic innervation of the BL were investigated by using an antibody against the vesicular acetylcholine transporter (VAChT). In KA-treated rats, it was found that (i) the BL shrunk to 25% of its original size (*p* < 0.01 vs. controls, Student’s *t*-test), (ii) the density of vesicular acetylcholine transporter-immunoreactive (VAChT-IR) varicosities was unchanged, (iii) the volumes of VAChT-IR cell bodies projecting to the BL from the horizontal limb of the diagonal band of Broca, ventral pallidum, and subcommissural part of the substantia innominata were significantly increased (*p* < 0.05, Bonferroni correction). These results illustrate significant changes in the basal forebrain cholinergic cells projecting to the BL in the presence of spontaneous recurrent seizures.

## 1. Introduction

Epilepsy is a neurological disease [1] affecting 65 million people around the world [2]. Associated with inherent comorbidities, this condition brings about decreased life expectancy, impaired life quality, loss of working years, and heavy healthcare costs [3,4,5,6,7]. The enduring tendency of generating spontaneous recurrent seizures, which characterizes epilepsy, is believed to be due to neuroplastic alterations at molecular, cellular, and circuitry levels, such as aberrant neurogenesis subsequent to the death of susceptible cell populations, rewiring, neuronal hyperexcitability and/or hypoexcitability, and other pathophysiological changes, including neuroinflammation and alterations in the extracellular matrix and blood–brain barrier [8,9,10,11,12]. Temporal lobe epilepsy (TLE) accounts for approximately 75% of epilepsy cases in adults, and is the most prevalent type of focal epilepsy in that population [13,14,15].

The administration of kainic acid (KA) to rodents [16,17,18,19,20] followed by status epilepticus (SE) represents one of the best characterized models of TLE, which, compared to other animal models, most closely mimics the behavioral, histopathologic, electrographic, and drug-response features of human TLE [21,22,23,24,25,26,27]. Previous works from our group have found that the KA model of TLE is characterized by neuroplastic alterations in cholinergic neurons of the medial septum and vertical limb of the diagonal band of Broca (MS/VDB) projecting to the hippocampal formation [28], as well as in the brainstem pedunculopontine and laterodorsal nuclei [29]. Since the amygdaloid complex, or more precisely its basolateral (basal) nucleus (BL), receives the densest cholinergic terminal projection in the central nervous system of both rodents and primates [30,31,32], and because various subdivisions of the BL undergo remarkable structural changes in epilepsy, we found it of interest to evaluate the effects of KA-induced epilepsy on the BL cholinergic afferents.

## 2. Results

### 2.1. Monitoring for Spontaneous Seizures

Two rats died acutely within 24 h following treatment with KA. During the first four weeks following KA injection, spontaneously recurrent stage 1–3 seizures on the Racine scale [21,33], that is, uncontrolled mouth and facial clonic movements and moderate forelimb clonus, were observed in only three rats from the KA group. However, in the remaining period of observation, all rats in the KA group displayed spontaneous stage 1–3 seizures. At least two spontaneous generalized behavioral seizures of stage 4–5 on the Racine scale were documented in all remaining KA-treated rats. Thus, the final group size for the KA-treated rats was *n* = 6. No behavioral seizures were observed in the control group (*n* = 6).

### 2.2. Basolateral Nucleus Volume

**Figure 1:**
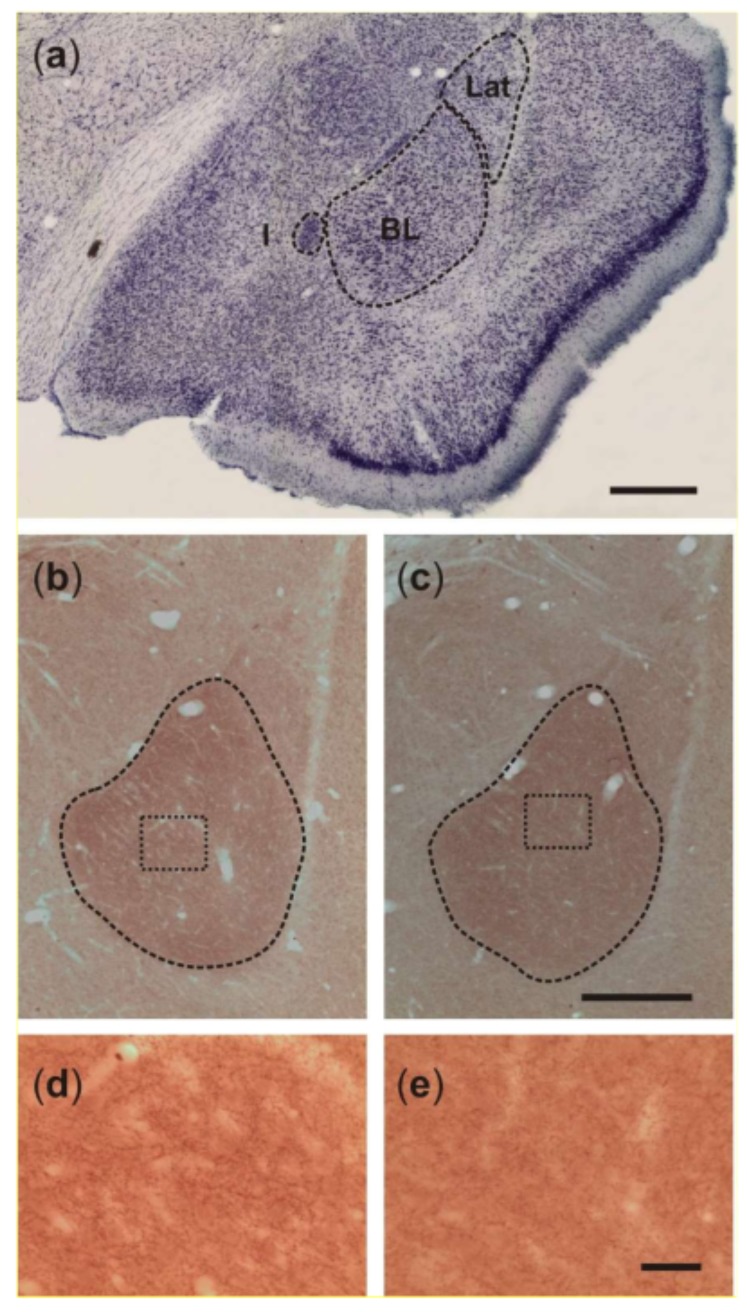
(**a**) Photograph of a representative Nissl-stained coronal section cut through the anterior part of the temporal lobe and showing the location of the basolateral nucleus (BL) relative to the neighboring lateral (Lat) and intercalated (I) nuclei. Scale bar, 200 μm. (**b**,**c**) Photomicrographs of representative vesicular acetylcholine transporter (VAChT)-stained sections obtained from control and post-SE rats, respectively. Note that the BL is more densely stained when compared to the lateral nucleus and other surrounding amygdaloid nuclei. The images are suggestive of moderate shrinkage of the BL in the post-SE rat. Scale bar, 200 μm. (**d**,**e**) Higher-power photomicrographs taken from the areas shown in insets (b,c), respectively. Note that the density of VAChT-stained fibers and fiber varicosities do not differ between the rats. Scale bar, 30 μm.

**Figure 2:**
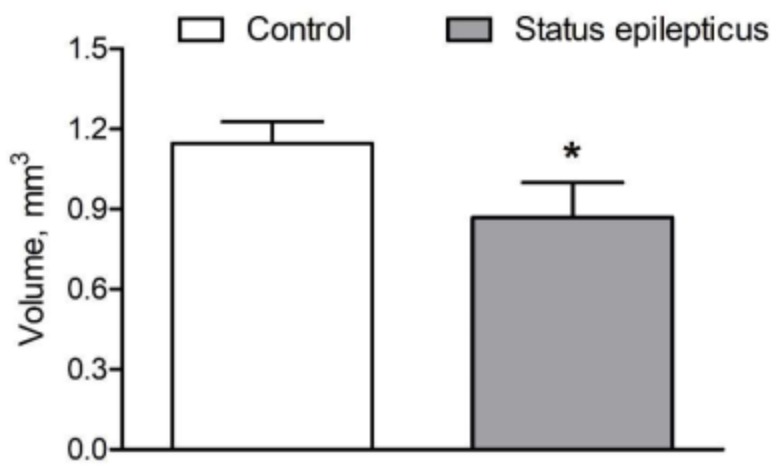
Effect of KA-induced status epilepticus (SE) on the total volume of the BL (mean ± standard deviation). Note that kainate treatment resulted in a 25% shrinkage of the BL as delineated in immunostained material (Figure 1). * *p* < 0.01, Student’s *t*-test.

Figure 1 shows representative images of level-matched sections cut through the BL and stained for vesicular acetylcholine transporter (VAChT) of a control rat and a KA-treated epileptic rat. As can be seen in these images, the epileptic state was associated with a decrease in volume of the BL. Volumes of the BL, estimated with an average coefficient of errors equal to 0.019, are shown in Figure 2 for both groups. Statistical comparisons of these estimates confirm significant shrinkage of the BL, approximately 25%, in KA rats, when compared to control rats (*p* < 0.01, Student’s *t*-test).

### 2.3. Density of VAChT-Immunoreactive Fiber Varicosities

**Figure 3:**
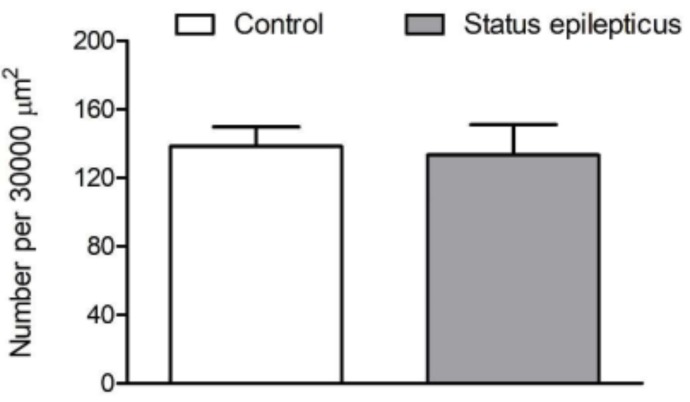
Graphic representation of the quantitative estimates obtained for the areal densities of vesicular acetylcholine transporter (VAChT)-stained varicosities in the BL of control and epileptic rats (see Figure 1). Values represent mean ± standard deviation. No differences between the groups were found.

Representative photomicrographs shown in Figure 1b–e illustrate VAChT-immunoreactive fibers organized in a dense network of terminals, which bear many varicosities of different shapes and sizes in the rat BL. However, the densities of the fibers and varicosities do not appear to differ considerably between control rats and post-SE rats. Figure 3 shows quantitative estimates of the areal density of VAChT-IR varicosities in the BL of control rats and post-SE rats. Both visual inspection of this plot and statistical analysis of the estimates (Student’s *t-*test) confirmed no differences between the two groups (*p* > 0.05).

### 2.4. Somatic Volume of VAChT-Immunoreactive Cells

**Figure 4:**
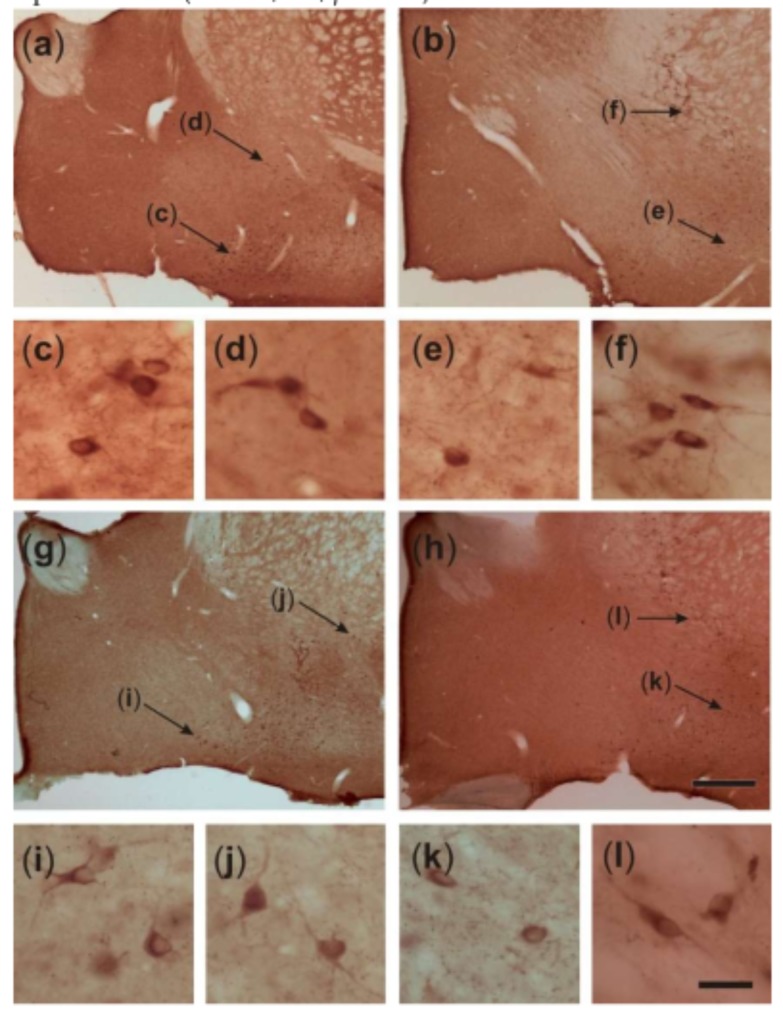
(**a**,**b**) Photomicrographs of representative vesicular acetylcholine transporter (VAChT)-stained coronal sections obtained from the brain of a control rat and showing four subdivisions of the basal forebrain projecting to the BL, horizontal limb of the diagonal band of Broca (HDB) (**c**), subcommissural part of substantia innominata (SI) (**d**), magnocellular preoptic nucleus (MCPO) (**e**), and ventral pallidum (VP) (**f**). The sections shown in (a,b) were cut approximately at levels of –0.72 and –1.20 mm, posterior to bregma, respectively (c–f). Higher-power photomicrographs of neurons indicated in (a,b) by arrows and belonging to the four basal forebrain subdivisions. (**g**,**h**) Photomicrographs of the respective VAChT-stained sections obtained from an epileptic rat. The sections (a–g,b–h) were cut at approximately the same levels relative to the bregma. (i–l) Higher-power photomicrographs of neurons found in the HDB, SI, MCPO, and VP of the epileptic rat. Precise locations of these neurons in the basal forebrain subdivisions are shown by arrows in (g,h). Note that neurons located in the HDB, SI, and VP of the epileptic rat possess larger perikarya than respective neurons of the control rat. Scale bars, 400 (**a**,**b**,**g**,**h**) and 30 μm (**c**–**f**,**i**–**l**).

**Figure 5:**
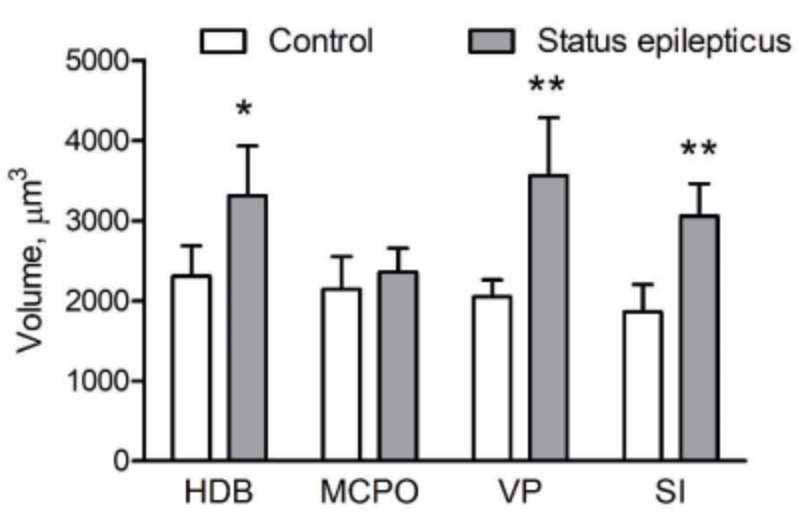
Graphic representation of stereological estimates for the mean somatic volume (mean ± standard deviation) of vesicular acetylcholine transporter-immunoreactive (VAChT-IR) cells in four distinct subdivisions of the basal forebrain (Figure 4). Note that post-SE rats have enlarged cholinergic neurons located in the horizontal limb of the diagonal band of Broca (HBD), ventral pallidum (VP), and substantia innominata (SI), but not in magnocellular preoptic nucleus (MCPO). * *p* < 0.05 and ** *p* < 0.005 vs the respective control region, Bonferroni correction.

Figure 4 shows representative microphotographs of VAChT-immunostained sections cut through the basal forebrain of a control rat (a,b) and a KA-treated epileptic rat (g,h). As can be inferred from the higher-power images in Figure 4c–f,i–l, respectively, the VAChT-IR cells of epileptic rats possess larger perikarya compared to respective cells of control rats. The mean somatic volumes of the VAChT-stained cells measured in four distinct areas of the basal forebrain are graphically represented in Figure 5. Multivariate analysis of variance (MANOVA) of these data yielded a significant main effect of treatment (Rao’s R_4,5_ = 6.65, *p* < 0.05). Bonferroni correction for multiple comparisons revealed that the perikarya of cholinergic cells were significantly enlarged in post-SE rats in the horizontal limb of the diagonal band of Broca (HDB; 44%, *p* < 0.05), ventral pallidum (VP; 75%, *p* < 0.005), and substantia innominata (SI; 66%, *p* < 0.005), but not in the magnocellular preoptic nucleus (MCPO; 9%, *p* > 0.05).

## 3. Discussion

The basolateral region of the amygdala is densely innervated by cholinergic afferents originating in different subdivisions of the basal forebrain, which suggests an important role of acetylcholine in modulating amygdala functions, including the processing of memory and emotion [34,35,36]. The main results of the present study are that, in epilepsy, this projection system undergoes severe modifications, which include significant decreases of that part of the amygdala that receives the densest cholinergic innervation, and abnormal increases of the perikarya of cells projecting to that part. Thus, it is likely that the changes in the amygdalopetal cholinergic connections to the basal forebrain described here may contribute to epilepsy-related hyperexcitability of amygdaloid neurons and comorbid deficits in amygdala-dependent behaviors.

Compared to the large body of data regarding the hippocampal pathology in TLE and respective animal models, the pathology of the amygdala in epilepsy has been relatively overlooked, having been addressed in just a few studies [37,38,39,40,41,42,43,44]. However, the amygdala is equally acknowledged as a brain area of very low ictal threshold, and kindling of the amygdala is one of the best known and characterized models of epilepsy [45,46]. Furthermore, in models where focal seizures are elicited in extra-amygdaloid areas, the amygdala plays a pivotal role in seizure spreading. For instance, in the audiogenic model of seizures, the ictal activity initiated in a primary brainstem focus (inferior colliculus of the mesencephalon) subsequently recruits the amygdala via the medial geniculate body prior to spreading to other forebrain structures [47,48,49]. Moreover, in human TLE, the amygdala can contain the main epilepsy focus in 5% of patients [50]. In the present study, we found that chronic epilepsy in rats is associated with dramatic shrinkage of the BL, which is consistent with the results of prior studies [41,51,52,53,54,55,56]. Similarly, magnetic resonance volumetric measurements of the amygdala in TLE have shown an average 20% volume reduction in 19% of the general population with TLE [57,58], and a 10%–30% volume reduction in patients with drug-refractory TLE [52,54]. Shrinkage of the amygdala in epilepsy is likely to result from neurodegenerative changes in this brain region, similar to those described in hippocampal sclerosis. Indeed, significant neuronal loss or reactive gliosis were reported in the basal [41,59] and lateral [41,60,61] nuclei of the amygdala in TLE patients. In animal models, some amygdala components, including the anterior cortical and medial nuclei, the medial division of the lateral nucleus, the parvicellular division of the basal nucleus, and the accessory basal nucleus were reported to be more susceptible to neuron loss with respect to other cerebral regions [37,39,41]. In addition, there is evidence that GABAergic interneurons are particularly vulnerable to seizure-induced damage [37,38,39,40,41,62], which leads to impaired feed-forward inhibition of principal amygdaloid neurons [63].

Cholinergic afferents play an important role in modulating the excitability of intrinsic neuronal circuits of the amygdala. The vast majority of cholinergic terminals form symmetric (putatively inhibitory) synapses on the dendritic shafts and spines of principal pyramidal neurons and on the perikarya of interneurons [64,65]. However, parts of the synapses possess characteristics of excitatory synapses [65] and principal cells of the amygdala are rich in both M1 (depolarizing) [66] and M2 (hyperpolarizing) [67] muscarinic receptors. Thus, it is plausible that cholinergic afferents have rather modulatory than inhibitory effects in the amygdala. Complementing previous experiments that illustrated an approximate 20% reduction in choline acetyltransferase activity [68], accompanied by a remarkable decrease in muscarinic receptor binding, we found that the areal density of cholinergic terminals was unchanged in the epileptic BL despite the considerable reduction of the total BL volume innervated by cholinergic fibers. Considered together with prior data showing preferential loss of GABAergic neurons in the amygdala of epileptic animals [37,38,39,62], our results suggest that induction of the chronic epilepsy state, at least in this model, alters the main pattern of cholinergic innervation in the BL. More specifically, it can be hypothesized that the cholinergic terminals, due to GABAergic cell loss, were relocated toward new targets, which could be dendritic and somatic membranes of pyramidal cells. This mechanism may be either compensatory, that is, aimed to provide additional inhibitory drive upon hyperexcitable BL principal neurons, or, in contrast, it may contribute to their hyperexcitability. Although further studies are necessary to address this issue in detail, the present findings clearly show that reorganization of the BL cholinergic network is likely to be one of the fundamental mechanisms underlying amygdala dysfunction in epilepsy.

These findings are consistent with our previous reports showing that KA-induced SE triggers marked reorganization of cholinergic afferents in the hippocampal formation, namely relocation of VAChT-containing terminals from the hilus of the dentate gyrus to its molecular layer [28,69]. This reorganization was found to be associated with a general activation of the septohippocampal cholinergic projection system, as indicated by the fact that cholinergic cells located in the MS/VDB regions projecting to the dentate gyrus of epileptic rats had approximately 40% larger cell bodies compared to control rats [28]. In this study, we estimated the somatic volumes of cholinergic cells in four distinct subdivisions of the basal forebrain, all of which project to the BL, although to different extents. In the HDB the cholinergic cells were enlarged by 44%, which is similar to what was previously found in the MS/VDB. Yet, even more profound hypertrophic changes were found in VAChT-IR cells located in the VP (75%) and SI (66%), suggesting that these regions are particularly implicated in epilepsy-related cholinergic plasticity. These data are compatible with the results of neuroanatomical studies showing that VP and SI neurons make a major contribution to cholinergic innervation of the amygdala [31,70,71]. Interestingly, neurons located in the MCPO, which send only a small number of cholinergic afferents to the amygdala, were not significantly enlarged. Although the importance of cholinergic plasticity in epilepsy is not yet clarified, the results of one study [72] indicate that site-specific inhibition of neuronal hypertrophy in the medial septum reduces seizure susceptibility and partially prevents epileptogenesis in a subset of KA-treated rats. Considered together with the present findings, these data suggest that a more widespread blockage of seizure-driven cholinergic hypertrophy, including in the medial septum and the regions examined in this study, might provide better protection against seizures and epileptogenesis.

In conclusion, the present study provides the first evidence of hypertrophic changes in the basal forebrain cholinergic cells projecting to the amygdala. These findings support the idea that epilepsy-related neuroplasticity of the forebrain cholinergic neurons can contribute to epileptogenesis as well as to epilepsy-related comorbid diseases. Thus, unraveling the biochemical pathways and physiological mechanisms that prompt hypertrophic changes in cholinergic neurons may offer new molecular targets for the development of novel therapeutic approaches to drug-resistant types of epilepsy.

## 4. Materials and Methods

### 4.1. Ethical Statement

The handling and care of animals was conducted according to the “Principles of laboratory animal care” (NIH publication No. 86–23, revised in 1985) [73] and Directive 2010/63/EU of the European Parliament. All experiments have been previously carefully evaluated and received approval by the Ethics Committee (Faculty of Medicine of Porto) and the General Veterinary Direction (3 April 2012) for the FCT application grant PTDC/SAU-NSC/115506/2009. Animals used were only those strictly required for the experiments and suffering was limited as much as possible.

### 4.2. Animals and Treatments

Ten-week-old male Wistar rats were randomly assigned to two groups, respectively, and given intraperitoneal (i.p.) injections of either kainic acid, 9.5 mg/kg, (KA group, *n* = 9) or the corresponding volume of saline (Ctrl group, *n* = 6). The animals were placed in individual boxes for behavioral observation and quantification for 6 h. Motor seizures were scored according to the modified Racine scale for generalized motor seizures of focal limbic initiation [21,33]. The onset of SE was defined as seizures scored as Racine’s stage 3–5, lasting for at least 20 min with no recovery of “baseline” behavior, such as grooming, sniffing, and exploratory activity [74]. One animal from the KA group exhibited numerous wet dog shake seizures, but did not reach Racine’s stage 3 or higher; this rat was excluded from the experiment. No seizure-like behavior was observed in rats given saline. Post-SE care consisted of evaluating the general state of animals twice a day, and giving subcutaneous saline injections, as well as moisturized chow from a plastic syringe. Starting from the second week after KA or vehicle treatments, the rats were daily monitored (except weekends) for the appearance of spontaneous motor seizures during at least two 2 h intervals (9:00–11:00 and 14:00–16:00) by an investigator blind to treatment groups.

### 4.3. Immunohistochemistry and Histology

Four months after the induction of SE, i.p. pentobarbital (90 mg/kg) was administered to animals to induce a deep anesthesia; then, animals were transcardially perfused with phosphate buffered saline (PBS), pH 7.4, 150 mL for vascular rinse, followed by paraformaldehyde 4% diluted in PBS, 250 mL for fixation. After removal, brains were immersed in fixative for 2 h and then immersed in 10% sucrose solution for 36 h at 4 °C. Afterwards, brains were vibratome-cut in 40 μm sections and stored in cryoprotectant (sucrose 30%, ethylene glycol 30%, polyvinylpirrolidone 0.25%, in PBS) at –20 °C until use.

Every sixth section, including basal forebrain and BL, was systematically sampled and immunostained for VAChT. Preceding primary antibodies, sections were rinsed in PBS (3 times) and endogenous peroxidases were blocked by immersing the sections in 1% H_2_O_2_ PBS solution for 15 min. Then, sections were washed in PBS (6 times, 5 min each) and blocked for nonspecific staining by immersion in 10% normal goat serum (NGS) + 0.5% Triton X-100 in PBS. Subsequently, sections were incubated in the presence of a VAChT guinea-pig primary antibody (Merck Millipore AB1588; 1:1250 dilution in PBS) for 72 h at 4 °C. After washing in PBS containing 2% NGS (3 times, 10 min each), sections were incubated with a biotinylated anti-guinea-pig antibody (BA7000, Vector Laboratories, Burlingame, CA 94010, USA, diluted 1:200 in 0.02 M PBS). Finally, sections were washed 3 times (10 min each) and incubated with avidin-biotin-peroxidase complex (Vector laboratories, Vectastain Elite ABC Kit) to perform the peroxidase reaction with 3,3’-diaminobenzidine (1 mg/mL) and H_2_O_2_ (0.08% in PBS). Further washing (2 times) with PBS preceded section mounting on gelatin-coated slides and overnight drying. For glass coverslipping, sections were dehydrated and covered with Histomount (National Diagnosis, Atlanta, GA, USA).

Nissl staining was performed using another set of regularly sampled sections (1:6), which were mounted on gelatin-coated slides and air-dried to be stained with Giemsa, dehydrated, and coverslipped with Histomount.

### 4.4. Density of VAChT-Immunoreactive Varicosities

Three to four consecutive VAChT-stained sections containing the BL were sampled from each brain. Sections were level-matched in all animals included in our analysis, by means of the rat brain atlas [75]. An Axio Scope.A1 microscope (Zeiss, Germany) equipped with a Leica EC3 color digital camera was used to visualize sections. BL boundaries were defined with a 10× objective lens on the basis of the rat brain atlas. For the purposes of the present study, we did not distinguish between the subdivisions of the BL nucleus [35,75]. In each section, four photomicrographs of the central area of the BL were taken, bilaterally, with a 100× objective lens, totaling 24–32 images per animal. Using the Fiji image-processing software (http://rsb.info.nih.gov/ij/) and a frame of 1421 µm^2^ superimposed onto each image, we measured all varicosities and their respective cross-sectional areas as found within the frame. Only fiber varicosities within cross-sectional areas ranging between 0.11 and 0.21 µm^2^ were then included for further analysis. Varicosities’ densities were normalized using an arbitrary area of 30,000 µm^2^ and averaged for all sections/animal.

### 4.5. Estimation of BL Volume

An Olympus BX-53 microscope equipped with a computer-controlled motorized stage system (MBF Bioscience, Williston, ND, USA) was used to visualize all sections containing the BL. The boundaries of the BL were consistently defined at all levels along the rostrocaudal axis using the rat brain atlas and the distinct pattern of BL VAChT staining, which is characteristic of this part of the amygdaloid complex. Estimations were carried out using the Cavalieri estimator probe of the Stereo Investigator software (MBF Bioscience). The regions of interest were delineated with a 4× objective lens and a grid size of 20 × 20 µm^2^ was applied.

### 4.6. Estimation of Somatic Volume of VAChT-Immunoreactive Neurons

VAChT-stained cells were sampled using an optical fractionator probe (Stereo Investigator software, MBF Bioscience, Williston, VT 05495, USA) to measure each somatic volume. The nucleator probe for isotropic systematically random sampled sections was used for measurements [76], and each cellular nucleolus was set as a central point. The estimates included the HDB, MCPO, VP, and the subcommissural part of SI. The ROIs were carefully outlined using a 10× objective lens, according to [75] for the HDB and MCPO, and [77] for the VP and SI. The coefficient of error for each individual estimate was inferior to 0.04 in all measurements for each animal.

### 4.7. Statistics

The volumes of cell bodies were analyzed using MANOVA followed by the Bonferroni post-hoc test for multiple comparisons (Bonferroni correction). The estimates of the BL volume and the density of VAChT-IR varicosities were analyzed by the two-tailed Student’s *t*-test. Results were presented as mean ± SD.

## Abbreviations

TLE	temporal lobe epilepsy
KA	kainic acid
SE	status epilepticus
LD	linear dichroism
MS	medial septum
VDB	vertical limb of the diagonal band of Broca
BL	basolateral amygdaloid nucleus
VAChT	vesicular acetylcholine transporter
HDB	horizontal limb of the diagonal band of Broca
VP	ventral pallidum
SI	substantia innominata
MCPO	magnocellular preoptic nucleus
PBS	phosphate buffered saline
NGS	normal goat serum
